# One-Pot Synthesis of β-Alanine from Fumaric Acid via an Efficient Dual-Enzyme Cascade Biotransformation

**DOI:** 10.3390/biom14121553

**Published:** 2024-12-05

**Authors:** Zifu Ni, Linshang Zhang, Azhen Nie, Huan Wang, Xiaoling Wu

**Affiliations:** 1 National Engineering Research Center of Wheat and Corn Further Processing, Henan University of Technology, Zhengzhou 450001, China; benzf@haut.edu.cn (Z.N.); lszhang@haut.edu.cn (L.Z.); 2 College of Biological Engineering, Henan University of Technology, Zhengzhou 450001, China; mxli@haut.edu.cn (A.N.); huwang@haut.edu.cn (H.W.); 3 College of Food Science and Engineering, Henan University of Technology, Zhengzhou 450001, China; 4 Laboratory of Applied Biocatalysis, School of Food Science and Engineering, South China University of Technology, No. 381 Wushan Road, Guangzhou 510640, China

**Keywords:** cascade reaction, methylaspartate ammonia-lyase, L-aspartate-α-decarboxylase, β-alanine, catalytic activity

## Abstract

As the only naturally occurring β-amino acid, β-alanine has important application prospects in many fields. Driven by the huge demand, biosynthesis is becoming more and more popular as a potential alternative to the chemical synthesis of β-alanine. Although the direct pathway from L-aspartic acid to β-alanine, catalyzed by L-aspartic acid-α-decarboxylase (PanD), is ideal for β-alanine synthesis, it is hindered by the high cost of the substrate and limited economic viability. In this work, a cell-free dual enzyme cascade system based on methylaspartate lyase (EcMAL) and panD was constructed to safely and efficiently synthesize β-alanine using fumarate as a substrate. Taking the previously engineered EcMAL as the target, CgPanD was finally screened as the best candidate through gene mining, sequence alignment, and enzyme property analysis. Finally, under the optimal conditions of 35 °C, pH 8.0, and EcMAL: CgPanD concentration ratio of 1:5, the yield of β-alanine reached 80% theoretical yield within 120 min. This study provides a potential strategy for the biosynthesis of β-alanine, paving the way for future industrial-scale production.

## 1. Introduction

β-Alanine is a natural β-amino acid synthesized by bacteria, fungi, and plants. Similarly to natural essential amino acids, animals need to obtain it from daily food [1]. Although β-alanine is an amino acid that is not involved in protein synthesis, it plays a variety of roles in the metabolism of animals, plants, and microorganisms. In animal nutrition, it primarily serves as a precursor for carnosine and anserine, active peptides derived from muscle tissue [2]. These peptides are crucial for enhancing muscle endurance and improving meat quality. Research indicates that β-alanine can boost animal productivity, modulate muscle growth, and regulate the levels of muscle-derived active peptides, thereby enhancing the quality of meat [3]. In plants, β-alanine is involved in the synthesis of pantothenic acid (Vitamin B5), a vital component of coenzyme A and acyl carrier proteins. These molecules are instrumental in plant metabolism [4]. Moreover, β-alanine has various plant-specific functions, such as acting as a stress response molecule that aids in resistance to extreme temperatures, hypoxia, drought, heavy metal toxicity, and certain biotic stresses [5]. Beyond its physiological functions, β-alanine and its derivatives have broad applications in medicine, the chemical industry, food additives, cosmetics, and even in environmental protection technologies like water treatment [6]. The expanding applications of β-alanine have led to a growing demand in the global market [7]. Currently, β-alanine production largely relies on chemical methods such as (1) conjugate addition of amine nucleophiles to α, β-unsaturated carboxylic acid derivatives, (2) hydrogenation of β-amino acrylates catalyzed by precious metals (Rh, Ir, Ru), and (3) multi-step Arndt–Eistert homologation reaction of α-amino acids and diazomethane (toxic and explosive) [8]. However, these methods usually require multiple steps of operation and purification of pre-functionalized substrates, so the step economy is low. They also often require harsh conditions such as high temperature or high pressure, which is inconsistent with the global green sustainable development concept [8]. Biosynthesis of β-alanine offers a promising alternative to chemical methods, overcoming their limitations. In recent years, significant advancements have been made in both yield and purity, positioning biological synthesis as a major breakthrough in the production of β-alanine [9].

Currently, the biosynthesis of β-alanine primarily employs three biological strategies: microbial fermentation, whole-cell biocatalysis, and enzyme catalysis [9,10]. Microbial fermentation leverages natural biosynthetic pathways to produce β-alanine, including the pyrimidine degradation pathway, aspartic acid pathway, spermine pathway, and propionic acid pathway [11]. These pathways have been introduced into model organisms such as Escherichia coli and yeast through synthetic biology strategies, enabling de novo synthesis of β-alanine using the host’s TCA cycle’s oxidative and reductive arms [12]. However, this approach necessitates extensive regulation during the construction of the de novo synthesis pathway, which can be operationally complex. Furthermore, the fermentation process often yields numerous by-products, complicating the separation and purification processes [13]. Additionally, the production of β-alanine can be achieved through the natural or heterologous expression of specific enzymes using whole cells of microorganisms, which is a widely utilized catalytic method [9,14]. One such method involves the one-step decarboxylation of L-aspartic acid to β-alanine, catalyzed by aspartic acid decarboxylase (ADC) [15]. Unfortunately, the high cost of the substrate L-aspartic acid and the substrate inhibition limits the industrialization of the method [7]. The enzymatic production of β-alanine has seen significant advancements with the expansion of protein engineering techniques. However, most of these methods rely on a single enzyme, such as ADC or PanD, or a combination of aspartic acid aminotransferase (AspA) with PanD, to catalyze the production of β-alanine from aspartic acid. Despite these advancements, the high costs associated with these methods remain a challenge [16]. In addition, the main advantages of the enzymatic synthesis method are the simple reaction mechanism, high yield, and high efficiency, while the main disadvantage is that the purification of AspA and ADC is expensive and easy to inactivate after extraction from cells. Therefore, the production cost of biocatalytic methods based on purified enzymes is a problem that needs to be solved for large-scale production.

Artificially designed enzyme cascades have gradually become an important alternative pathway for the production of fine chemicals [17]. Given the high chemical and stereoselectivity typically required for fine chemicals, enzyme cascades can circumvent the need for separation and purification of reaction intermediates and by-products, thereby reducing costs and enhancing production efficiency [18]. Furthermore, enzyme cascades can surpass the limitations of the reaction’s equilibrium, prevent the formation of unstable or toxic intermediates, and facilitate the conversion of initial substrates to final products [19]. Consequently, enzyme cascades often achieve higher yields than traditional single-step transformations. However, a critical challenge in enzyme cascades is the efficient synergy of enzymes with distinct characteristics within a one-pot environment [20]. Addressing this challenge necessitates a systematic approach to substrate compatibility, environmental conditions, and enzyme interactions, as well as precise control over variables during the optimization process. To construct an efficient enzyme cascade system, various strategies have been developed to address adaptation issues within the reaction process [21]. On the one hand, gene mining strategies can identify potential protein sequences with desired properties from databases or target hosts, with their reaction performance subsequently tested through actual construction [22]. For instance, a novel ω-transaminase with robust biological activity at lower pH levels was discovered through genome database mining [23]. The epoxyhydrolase, alcohol dehydrogenase, and transaminase were expressed by recombinant *E. coli*, and an efficient cascade reaction was constructed [24]. On the other hand, the key enzymes can be replaced on the basis of the original cascade reaction. The replacement method can be to screen the isozymes with better performance, or to strengthen the existing enzymes by engineering methods. In the previous study of methylaspartate lyase, the natural methylaspartate lyase had the defect of poor stability [25,26]. The author introduced the mutation site to construct the disulfide bond, which increased its thermal stability by about 26 times, thus expanding its application range [25]. Additionally, other derivative methods in enzyme cascades, such as immobilization, have garnered widespread attention [12]. The implementation of these methods fundamentally aims to increase the stability and efficiency of the cascade.

In our previous studies, a novel methylaspartate lyase (EcMAL) was identified from *E. coli* O157:H7, which exhibits highly active and perfect enantiomeric selectivity in the hydroamination addition reaction utilizing short-chain unsaturated acids as substrates [27]. However, a significant decrease in reaction activity was observed when the temperature surpassed the optimal range, adversely affecting the construction of our subsequent cascade reaction system. To achieve the objective of synthesizing higher-value fine chemicals from the inexpensive substrate, fumaric acid, we engineered a two-enzyme cascade reaction system in this study, relying on EcMAL and PanD. To enhance the efficiency of the cascade reaction, we employed the reaction conditions of methylaspartate lyase as a benchmark to explore and screen three distinct sources of PanDs. Subsequently, we optimized the reaction conditions of the system to improve both the yield and synthesis efficiency of β-alanine.

## 2. Materials and Methods

### 2.1. Bacterial Strains, Plasmids, and Chemicals

*E. coli* BL21 (DE3) was purchased from TransGen Biotech Co., Ltd. (Beijing, China) and used for protein production. For cloning, *E. coli* DH5α was obtained from Sangon Biotech Co., Ltd. (Shanghai, China). The genes encoding for *panD* from *Corynebacterium glutamicum*, *E. coli*, and *Thermus thermophiles* were obtained from NCBI. The obtained sequences were codon optimized for *E. coli* from General Biology Co., Ltd. (Chuzhou, China) and cloned into pET-32a vectors via EcorI/HindIII restriction sites. The recombinant plasmids pET-32a-CgPanD, pET-32a-EcPanD, and pET-32a-TtPanD were all synthesized and constructed by General Biotechnology Co., Ltd. The plasmid (pET-32a-EcMAL) coding for methylaspartate lyase from *E. coli* O157:H7 has been prepared previously in our lab [27]. All sequences can be found in the supplement. The Luria–Bertani (LB) medium components, Terrific Broth (TB) medium, ampicillin (Amp), isopropyl-β-d-thiogalacto-side (IPTG), and other chemicals were obtained from Aladdin (Shanghai, China). Fumaric acid, aspartic acid, and β-alanine were purchased from Sigma-Aldrich (St. Louis, MO, USA), and were of analytical or higher grade.

### 2.2. Protein Expression and Purification

The recombinant plasmids pET-32a-CgPanD, pET-32a-EcPanD, and pET-32a-TtPanD were cultured overnight in *E. coli* BL21 (DE3) by LB medium in shake flask culture, supplemented with 50 µg/mL Amp at 37 °C and 200 rpm. The strain seed liquid was then inoculated with TB medium at a concentration of 1% inoculum until the OD600 reached 0.6–0.8. Then, the IPTG was added to induce protein expression at a final concentration of 0.1 mM. The expressions were carried out overnight at 16 °C and 180 rpm, and the wet cells were harvested by centrifuging at 4 °C and 8000× *g* for 15 min. Cells were gently resuspended in 100 mM Tris-HCl buffer pH 7.5 and then subjected to ultrasonication while maintained on ice to ensure optimal activities. The lysates were clarified by centrifugation to remove debris. The enzymes were subsequently loaded onto a HisTrap Ni-NTA FF column (GE Healthcare, Chicago, IL, USA), which had been pre-equilibrated with a buffer consisting of 100 mM Tris at pH 7.5, supplemented with 25 mM imidazole and 500 mM NaCl, in accordance with the manufacturer’s guidelines. The bound proteins were eluted using a buffer containing 250 mM imidazole. To remove any residual salt, the eluted proteins were desalted using a Hi Prep 26/10 desalting column (GE Healthcare) that had been equilibrated with 100 mM Tris buffer at pH 7.5. The purified proteins were then flash-frozen at −80 °C to preserve their integrity. For each experimental use, the proteins were freshly thawed to ensure optimal activity. The purified enzyme was verified by SDS-PAGE and the protein concentration was determined by the Bradford method.

### 2.3. Enzyme Assay

The single-step enzyme activity needs to be detected in the enzyme activity analysis of cascade reaction. In the enzyme cascade reaction of β-alanine synthesis, the activity detection method of EcMAL has been described in detail in previous studies [25]. Here, the enzyme activity determination procedure of PanDs is described. To ensure the high efficiency of the cascade reaction, the enzyme activity determination conditions of PanDs are the same as EcMAL. The reaction was performed in a 5 mL reaction system containing 20 mM aspartic acid as substrate in 500 mM Tris-HCl buffer (pH 8.0), 500 mM NH_4_Cl, and 20 mM MgCl_2_. In all reaction systems, the purified enzyme concentration was set at 0.1 mg/mL. The enzymatic reaction was meticulously conducted at a controlled temperature of 30 °C with a gentle agitation rate of 180 rpm. At precise 5 min intervals, aliquots of the reaction mixture were withdrawn to monitor the progress of the reaction. To terminate the reaction, the samples were subjected to heat inactivation by immersing them in boiling water for a brief period of 5 min. Subsequently, the treated samples were analyzed using high-performance liquid chromatography (HPLC) to determine the reaction products and their concentrations. To ensure the reliability and reproducibility of the results, each experiment was performed in triplicate, thereby providing a robust statistical basis for the analysis. One unit (U) of PanDs activity were defined as the amount of enzyme consuming 1 mmol/min of substrate at 35 °C and pH 8.0.

### 2.4. Analytical Methods

The concentration of fumaric acid, L-aspartic acid, and β-Ala were determined by HPLC with a Chiral AAOA column (5 μm, 4.6 × 150 mm). The analysis was performed at 30 °C with mobile phase A:2 mM solution of CuSO_4_, mobile phase B: isopropanol, at a flow rate of 1.0 mL/min (mobile phase A:B = 95:5), and the analytes were detected at 254 nm. Standard curves were made with substrates and products of different concentration gradients.

### 2.5. Biochemical Characterization

The optimal pH of aspartic acid α-decarboxylase was determined using L-aspartic acid as the reaction substrate. At 30 °C, the enzyme activity was investigated in the range of pH 3.0–9.0, and the reaction system was consistent with the enzyme activity determination system. Different pH ranges were replaced with different buffers: pH 3.0–7.0: phosphate-citric acid and pH 7.0–9.0: Tris-HCl buffer. The effect of pH on the activity of aspartic acid α-decarboxylase was expressed as relative enzyme activity, and the maximum activity was defined as 100%. The optimal temperature of aspartic acid α-decarboxylases was measured under temperatures ranging from 25 to 60 °C at the optimal pH. The optimum temperature and thermostability of the enzyme were obtained by plotting the relative enzyme activity against temperature. All original data were expressed as mean ± standard deviation (SD) obtained from at least triplicated experiments.

### 2.6. Kinetic Assay

The kinetic parameters of PanDs were determined by measuring the initial velocities of the enzymatic reaction and curve-fitting according to the Michaelis−Menten equation using GraphPad 7.0 software. The enzyme activity for decarboxylation was obtained by HPLC. The reaction was carried out in Tris-HCl buffer (500 mM, pH 8.0) with 500 mM NH_4_Cl and 20 mM MgCl_2_. The substrate concentration range was between 1 and 20 mM. Kinetic constants *K_m_* and *V_max_* were determined by Lineweaver-Burk plots.

### 2.7. Cascade Reactions

The purified EcMAL and CgPanD were diluted to the same concentration. The reaction was performed in a 5 mL reaction system containing 20 mM fumaric acid as substrate in 500 mM Tris-HCl buffer (pH 8.0), 500 mM NH_4_Cl, and 20 mM MgCl_2_. The cascade reaction starts with the addition of an enzyme solution with a concentration ratio of 1:3, 1:5, and 1:7, at 35 °C and 180 rpm. Samples were taken at intervals and assayed using the enzyme assay described above. Each experiment was performed in triplicate.

## 3. Results and Discussion

### 3.1. Design the Cascade Reaction by EcMAL and PanDs

**Figure 1:**
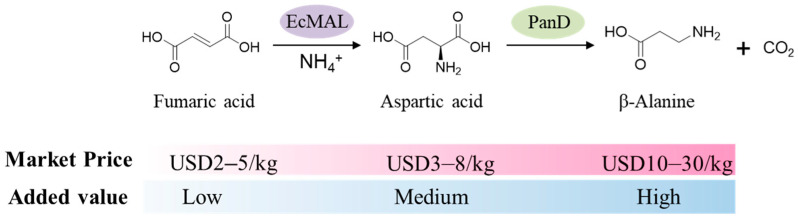
Design and construction of a dual-enzyme cascade synthesis pathway for β-alanine via EcMAL and PanD, using fumarate as the initial substrate. Their market prices and added values were also compared.

Previous studies have shown that EcMAL has good catalytic activity and high enantiomeric selectivity in the process of catalyzing unsaturated dicarboxylic acids as natural substrates, especially in the synthesis of L-aspartic acid from fumaric acid as substrate, showing 83% of the target product with perfect enantiomeric excess (≥99%) [27]. However, EcMAL is greatly affected by reaction conditions and, therefore, requires stringent reaction conditions. On this basis, we tried to extend the reaction steps by inserting other enzymes, and then obtained other more value-added amino acids and their derivatives with fumaric acid as the starting point. Considering that there are two asymmetric carboxylic acid structures in the chemical structure of aspartic acid, L-alanine and β-alanine can be generated after an asymmetric decarboxylation reaction. They have important applications in the fields of food, medicine, and fine chemicals, respectively. Since β-alanine is relatively expensive in the market, and when fumaric acid is used as a substrate, the price of fumaric acid is lower than that of the intermediate products L-aspartic acid and β-alanine, this reaction has commercial value. Therefore, with the synthesis of β-alanine as the goal, a dual enzyme cascade scheme was designed. The first step in this scheme is to convert fumaric acid into L-aspartic acid through the hydroamination addition reaction of EcMAL. Subsequently, PanD catalyzed the α-decarboxylation of L-aspartic acid to produce β-alanine and CO_2_ (Figure 1). In theory, this cascade reaction can produce one molecule of β-alanine with one molecule of fumaric acid, and the by-product is CO_2_, which facilitates the subsequent product separation.

In order to construct a free two-enzyme cascade system, we used the amino acid sequence of PanD from Bacillus subtilis as a probe to perform BLAST analysis in the Uniprot protein database [7]. The PanD genes from 20 different sources were selected and the phylogenetic tree was constructed by the maximum likelihood method in MEGA 10 software, and the genetic relationship between different sequences was analyzed (Figure S1). Among them, because the research on BsPanD has been more extensive and in-depth, we chose the sequence with a large difference when selecting the candidate sequence. The sequences with similar genetic relationships have certain similarities in structure and function, so they are selected from Corynebacterium glutamicum and Escherichia coli. In addition, considering that the enzymes in thermotolerant host bacteria have natural thermal stability properties, Thermus thermophilus was selected as a candidate. The PanD sequences of the above three sources were analyzed for homology with the PanD sequences of Bacillus subtilis. In order to understand the sequence characteristics of PanD from the three sources more specifically, multiple sequence alignments were performed on them, as shown in Figure S2. The results of the key residues showed that the PanD genes from the three sources all have highly conserved Gly24-Ser25, so they can form active acetone acyl groups [28]. The 54 position is arginine, which can specifically recognize the substrate L-aspartic acid; the 58 position is tyrosine, as a proton donor of the enol structure in the catalytic process, it can form an enzyme-product Schiff base intermediate [14]. Therefore, these three PanDs from different sources have the potential to catalyze L-aspartate to produce β-alanine, and were preferentially selected as candidates.

### 3.2. Optimization of Reaction Conditions for PanDs

**Figure 2:**
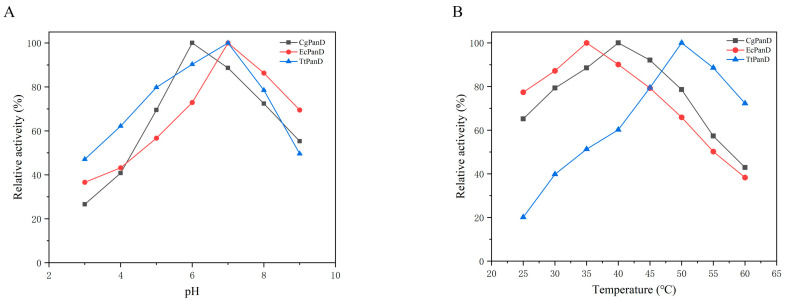
(**A**) The optimum reaction pH of PanDs. Buffers: phosphate-citric acid (pH 3.0–7.0), Tris-HCl buffer (pH 7.0–9.0). (**B**) The optimum reaction temperature of PanDs. The reaction used L-aspartic acid as the substrate, and the maximum activity was defined as 100%.

In order to eliminate the effect of protein expression on the evaluation of enzyme activity in cell reaction, the catalytic ability of PanD from three sources was accurately compared, and the activity of the pure enzyme was determined by purifying the recombinant enzyme. Considering that the sources of the enzymes are different and may have different catalytic characteristics, the optimum conditions of the three enzymes were first investigated with L-aspartic acid as the substrate. As shown in Figure 2A, the optimum pH of CgPanD was 6.0, while EcPanD and TtPanD showed the highest activity at pH 7.0. This result is similar to other reported PanDs, most of which belong to neutral and acidic enzymes [18]. Moreover, once they deviated from the optimum pH, their activities decreased significantly. Considering that the first step of the reaction catalyzed by EcMAL needs to be carried out at pH 8.0, and the three PanDs retain more than 60% relative activity at this pH. Although PanDs retain most of their activity at this pH, if they want to reach the scale of industrial production, they need to be strengthened by subsequent methods such as immobilization or enzyme engineering. In addition, the optimum temperature of the three PanDs at the optimum pH was also explored. As shown in Figure 2B, the optimum temperature of the three PanDs is quite different. Because TtPanD is derived from thermophilic bacteria, it reaches the maximum at 50 °C, while the optimum temperature of CgPanD and EcPanD is 35 °C and 40 °C, respectively. The relative activity of CgPanD and EcPanD remained above 60% between 25 °C and 45 °C, indicating that they have better adaptability than TtPanD in the cascade reaction with EcMAL (optimum temperature 30 °C).

### 3.3. Comparison of the Activity of Aspartic Acid α-Decarboxylase from Different Sources

**Figure 3:**
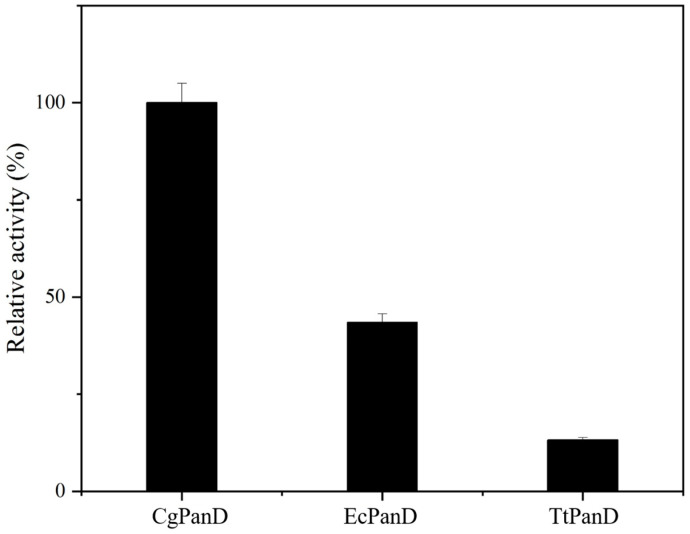
The activity comparison of PanDs. The decarboxylation reaction of the PanDs were carried out under their respective optimal conditions, with the activity of CgPanD defined as 100%, and the activity of EcPanD and TtPanD were expressed as relative activity.

The determination of the kinetic constants of the enzymatic reaction can more intuitively understand the affinity of different PanDs to the substrate L-aspartic acid. There is a substrate inhibition phenomenon in the catalytic process of PanDs, and the enzyme activity will decrease with increasing substrate concentration. Therefore, the enzyme kinetic constants were determined with L-aspartic acid at final concentrations of 1, 2, 5, 10, and 20 mM. As shown in Table 1, CgPanD from *Corynebacterium glutamicum* has the lowest *K_m_* of 4.3 mmol/L, indicating that the enzyme has a high affinity with the substrate L-aspartate sodium and is more easily combined with the substrate, which is also the reason why its enzyme activity is higher than other sources. The *k_cat_* value was the largest, 2.8 s^−1^, indicating that a single enzyme molecule converted a single substrate molecule quickly. In addition, in order to ensure the synergy of the cascade reaction formed by EcMAL and PanDs, the enzymatic properties of EcMAL and the optimum conditions of PanDs were conjugated, and the reaction was finally determined at 35 °C and pH 8.0. The activity comparison of the three PanDs under this condition is shown in Figure 3, the relative activity was calculated with the best PanD activity as 100%, indicating that CgPanD has higher activity than the other two PanDs under this condition. Therefore, CgPanD was selected for the construction of the free two-enzyme cascade system.

### 3.4. Construction and Optimization of the Free Two-Enzyme Cascade Reaction

**Figure 4:**
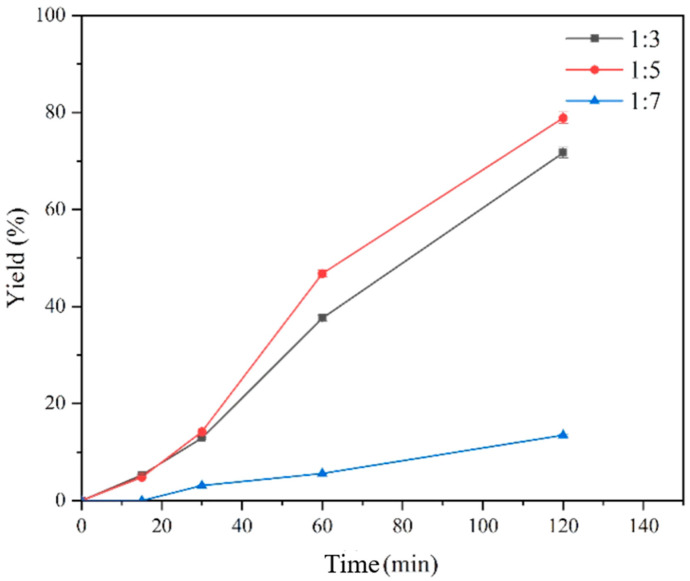
Optimization of enzyme concentrations for free enzyme cascades composed of EcMAL and CgPanD. The concentration ratios of EcMAL to CgPanD were 1:3, 1:5, and 1:7. The reaction conditions were 20 mM fumaric acid, 500 mM Tris-HCl buffer (pH 8.0), 500 mM NH_4_Cl, and 20 mM MgCl_2_ at 30 °C.

To further construct a free double enzyme cascade reaction between the screened aspartic acid α-decarboxylase CgPanD and EcMAL, we used fumaric acid as a substrate to investigate the rate-limiting step of the free double enzyme and the concentration ratio of the double enzyme. The enzyme kinetic parameters and enzyme activity of EcMAL and CgPanD showed that the rate-limiting step in the cascade was the decarboxylation of aspartic acid. Among them, the enzyme activity of EcMAL is about eight times that of CgPanD. Considering the synergy of the cascade reaction, the concentration ratio of EcMAL to CgPanD was set to 1:3, 1:5, and 1:7 for optimization based on the comparison of the enzyme activities of the two. Under the reaction conditions with 20 mM fumarate as the substrate, as shown in Figure 4, when the concentration ratio of EcMAL to CgPanD was 1:5, the highest yield of β-alanine reached about 80% of the theoretical yield within 120 min of the same reaction time, which was seven times higher than that at 1:7. The reason for this may be that there may be some inhibitors in PanD, and the dosage effect at high concentrations hinders the cascade reaction. Compared with the single enzyme reaction directly using aspartic acid as the substrate, the free double enzyme cascade has obvious advantages, especially in the high conversion efficiency with lower fumaric acid as the substrate.

## 4. Conclusions

In constructing a dual-enzyme cascade reaction system, enzymes sourced from different origins exhibit distinct enzymatic properties. Consequently, enzyme compatibility is crucial for influencing the efficiency of cascade catalysis. Aiming to achieve one-pot dual-enzyme catalysis of fumaric acid to β-alanine, this study commenced with an analysis of the enzymatic characteristics of EcMAL, which was previously obtained. Through gene mining and sequence analysis, three distinct sources of aspartate α-decarboxylase PanD were identified as potential candidates. Utilizing the reaction conditions of EcMAL as a benchmark, subsequent optimization and evaluation of enzyme kinetic parameters led to the identification of CgPanD as the enzyme with optimal compatibility with EcMAL. The ratio for the dual-enzyme cascade reaction was fine-tuned based on the catalytic performance of both enzymes, culminating in the determination that a concentration ratio of EcMAL to CgPanD of 1:5 yielded 80% β-alanine within 120 min. This research not only expands the potential applications of EcMAL but also establishes a foundation for a novel cascade pathway in β-alanine synthesis.

## Figures and Tables

**Table 1:** Kinetic constants of EcMAL and CgPanD.

Parameters	EcMAL	CgPanD	EcPanD	TtPanD
*K_m_* (mM)	6.7 ± 0.1	4.3 ± 0.2	7.9 ± 0.1	8.5 ± 0.1
*K_cat_* (s^−1^)	87 ± 3.8	2.8 ± 0.5	1.4 ± 0.2	2.2 ± 0.1
*K_cat_*/*K_m_* (s^−1^∙mM^−1^)	13	0.65	0.18	0.26
Specific enzyme activity (U mg^−1^ protein)	82 ± 3.6	10.5 ± 2.8	2.3 ± 0.1	2.8 ± 0.1

## Data Availability

The original contributions presented in the study are included in the article and Supplementary Materials, further inquiries can be directed to the corresponding authors.

## Supplementary Materials

The following supporting information can be downloaded at: https://www.mdpi.com/article/10.3390/biom14121553/s1, Figure S1. Phylogenetic tree of PanDs.; Figure S2. Multiple sequence alignment results of PanDs [29,30,31,32,33].

## References

[1] Lu J., Wang G., Yang C., Peng Z., Yang L., Du B., Guo C., Sui S., Wang J., Li J. (2023). Study on the Construction Technology of β-Alanine Synthesizing *Escherichia coli* Based on Cellulosome Assembly. Front. Bioeng. Biotechnol..

[2] Shen Y., Zhao L., Li Y., Zhang L., Shi G. (2014). Synthesis of β-Alanine from l-Aspartate Using l-Aspartate-α-Decarboxylase from *Corynebacterium glutamicum*. Biotechnol. Lett..

[3] Meenukutty M.S., Mohan A.P., Vidya V.G., Viju Kumar V.G. (2022). Synthesis, Characterization, DFT Analysis and Docking Studies of a Novel Schiff Base Using 5-Bromo Salicylaldehyde and β-Alanine. Heliyon.

[4] Lu S., Zhou C., Guo X., Du Z., Cheng Y., Wang Z., He X. (2022). Enhancing Fluxes through the Mevalonate Pathway in *Saccharomyces* Cerevisiae by Engineering the HMGR and β-Alanine Metabolism. Microb. Biotechnol..

[5] Poon N.Y., Sinskey A.J., Zhou K. (2023). Engineering *Escherichia coli* to Assimilate β-Alanine as a Major Carbon Source. Appl. Microbiol. Biotechnol..

[6] de Salazar L., Segarra I., López-Román F.J., Torregrosa-García A., Pérez-Piñero S., Ávila-Gandía V. (2021). Increased Bioavailability of β-Alanine by a Novel Controlled-Release Powder Blend Compared to a Slow-Release Tablet. Pharmaceutics.

[7] Xu J., Zhou L., Yin M., Zhou Z. (2021). Novel Mode Engineering for β-Alanine Production in *Escherichia coli* with the Guide of Adaptive Laboratory Evolution. Microorganisms.

[8] Tan G., Das M., Keum H., Bellotti P., Daniliuc C., Glorius F. (2022). Photochemical Single-Step Synthesis of β-Amino Acid Derivatives from Alkenes and (Hetero)Arenes. Nat. Chem..

[9] Wang J., Ma D., Mai D., Li H., Wang J., Wang X., Chen K., Ouyang P. (2022). β-Alanine Production by L-Aspartate-α-Decarboxylase from *Corynebacterium glutamicum* and Variants with Reduced Substrate Inhibition. Mol. Catal..

[10] Yuan S.F., Nair P.H., Borbon D., Coleman S.M., Fan P.H., Lin W.L., Alper H.S. (2022). Metabolic Engineering of *E. coli* for β-Alanine Production Using a Multi-Biosensor Enabled Approach. Metab. Eng..

[11] Ghiffary M.R., Prabowo C.P.S., Adidjaja J.J., Lee S.Y., Kim H.U. (2022). Systems Metabolic Engineering of *Corynebacterium glutamicum* for the Efficient Production of β-Alanine. Metab. Eng..

[12] Zou S.M., Wang J.P., Zong M.H., Wang Z.L., Zheng Z.J., Li N. (2023). One-Pot Photoenzymatic Synthesis of Maleic Acid and Its Derivatives from Bio-Based Furfural via Catalytic Cascades. Green Chem..

[13] Yu X.J., Huang C.Y., Xu X.D., Chen H., Liang M.J., Xu Z.X., Xu H.X., Wang Z. (2020). Protein Engineering of a Pyridoxal-50-Phosphate-Dependent l-Aspartate-α-Decarboxylase from Tribolium Castaneum for β-Alanine Production. Molecules.

[14] Hu Z.C., Tian Y.H., Yang J.L., Zhu Y.N., Zhou H.Y., Zheng Y.G., Liu Z.Q. (2023). Research Progress of L-Aspartate-α-Decarboxylase and Its Isoenzyme in the β-Alanine Synthesis. World J. Microbiol. Biotechnol..

[15] Wang L., Piao X., Cui S., Hu M., Tao Y. (2020). Enhanced Production of β-Alanine through Co-Expressing Two Different Subtypes of l-Aspartate-α-Decarboxylase. J. Ind. Microbiol. Biotechnol..

[16] Miao L., Li Y., Zhu T. (2021). Metabolic Engineering of Methylotrophic Pichia Pastoris for the Production of β-Alanine. Bioresour. Bioprocess..

[17] Wu J., Ma B.-D., Xu Y. (2023). One-Pot Synthesis of β-Alanine from Maleic Acid via Three-Enzyme Cascade Biotransformation. Catalysts.

[18] Fan A., Li J., Yu Y., Zhang D., Nie Y., Xu Y. (2021). Enzymatic Cascade Systems for D-Amino Acid Synthesis: Progress and Perspectives. Syst. Microbiol. Biomanuf..

[19] Sperl J.M., Sieber V. (2018). Multienzyme Cascade Reactions—Status and Recent Advances. ACS Catal..

[20] Noordzij G.J., Wilsens C.H.R.M. (2019). Cascade Aza-Michael Addition-Cyclizations; Toward Renewable and Multifunctional Carboxylic Acids for Melt-Polycondensation. Front. Chem..

[21] Liu R., Wang J., Xiong P., Chen Q., Liu H. (2021). De Novo Sequence Redesign of a Functional Ras-Binding Domain Globally Inverted the Surface Charge Distribution and Led to Extreme Thermostability. Biotechnol. Bioeng..

[22] Liu Q., Xun G., Feng Y. (2019). The State-of-the-Art Strategies of Protein Engineering for Enzyme Stabilization. Biotechnol. Adv..

[23] Zhang T., Zhang R., Xu M., Zhang X., Yang T., Liu F., Yang S., Rao Z. (2018). Glu56Ser Mutation Improves the Enzymatic Activity and Catalytic Stability of *Bacillus subtilis* L-Aspartate α-Decarboxylase for an Efficient β-Alanine Production. Process Biochem..

[24] He F.S., Jin J.H., Yang Z.T., Yu X., Fossey J.S., Deng W.P. (2016). Direct Asymmetric Synthesis of β-Bis-Aryl-α-Amino Acid Esters via Enantioselective Copper-Catalyzed Addition of p-Quinone Methides. ACS Catal..

[25] Ni Z.-F., Xu P., Zong M.-H., Lou W.-Y. (2021). Structure-Guided Protein Engineering of Ammonia Lyase for Efficient Synthesis of Sterically Bulky Unnatural Amino Acids. Bioresour. Bioprocess.

[26] Ni D., Zhang S., Klrtel O., Xu W., Chen Q., Öner E.T., Mu W. (2021). Improving the Thermostability and Catalytic Activity of an Inulosucrase by Rational Engineering for the Biosynthesis of Microbial Inulin. J. Agric. Food Chem..

[27] Ni Z.F., Zeng Y.J., Xu P., Guo Z.W., Ou X.Y., Peng F., Yang J.G., Zong M.H., Lou W.Y. (2020). Characterization of a Novel Methylaspartate Ammonia Lyase from *E. coli* O157:H7 for Efficient Asymmetric Synthesis of Unnatural Amino Acids. ACS Sustain. Chem. Eng..

[28] Àvila-Cabré S., Pérez-Trujillo M., Albiol J., Ferrer P. (2023). Engineering the Synthetic β-Alanine Pathway in Komagataella Phaffii for Conversion of Methanol into 3-Hydroxypropionic Acid. Microb. Cell Fact..

[29] Lambrughi M., Maršić Ž.S., Saez-Jimenez V., Mapelli V., Olsson L., Papaleo E. (2019). Conformational Gating in Ammonia Lyases. bioRxiv.

[30] Cheng X., Chen X., Feng J., Wu Q., Zhu D. (2018). Structure-Guided Engineering of: Meso -Diaminopimelate Dehydrogenase for Enantioselective Reductive Amination of Sterically Bulky 2-Keto Acids. Catal. Sci. Technol..

[31] Yuan S., Chan H.C.S., Filipek S., Vogel H. (2016). PyMOL and Inkscape Bridge the Data and the Data Visualization. Structure.

[32] Yu S., Yao P., Li J., Feng J., Wu Q., Zhu D. (2019). Improving the Catalytic Efficiency and Stereoselectivity of a Nitrilase from: Synechocystis Sp. PCC6803 by Semi-Rational Engineering En Route to Chiral γ-Amino Acids. Catal. Sci. Technol..

[33] Wang J.B., Lonsdale R., Reetz M.T. (2016). Exploring Substrate Scope and Stereoselectivity of P450 Peroxygenase OleTJE in Olefin-Forming Oxidative Decarboxylation. Chem. Commun..

