# Supplementary material for: One-Pot Synthesis of β-Alanine from Fumaric Acid via an Efficient Dual-Enzyme Cascade Biotransformation
Source: Biomolecules. 2024 Dec 5;14(12):1553. doi: 10.3390/biom14121553 (PMC11674828; doi:10.3390/biom14121553)
Supplement: Supplementary file 1 [file biomolecules-14-01553-s001.zip › biomolecules-3257194-supplementary.pdf]

Supporting Information

One-Pot Synthesis of β-Alanine from Fumaric Acid Via an Efficient Dual-Enzyme Cascade Biotransformation

Zifu Ni[1,2], Linshang Zhang[1,3], Azhen Nie[2], Huan Wang[2], Xiaoling Wu[1,4]*

[1] National Engineering Research Center of Wheat and Corn Further Processing, Henan University of Technology, Zhengzhou, 450001, China

[2] College of Biological Engineering, Henan University of Technology, Zhengzhou, 450001, China

[3] College of Food Science and Engineering, Henan University of Technology, Zhengzhou, 450001, China

[4] Laboratory of Applied Biocatalysis, School of Food Science and Engineering, South China University of Technology, No. 381 Wushan Road, Guangzhou 510640, China

* Corresponding author: wux118@scut.edu.cn (W.X.)

Contents

1 Supporting Figures **3**

Figure S1. Phylogenetic tree of PanDs. 3

Figure S2. Multiple sequence alignment results of PanDs. 4

2. References: **5**

1 Supporting Figures

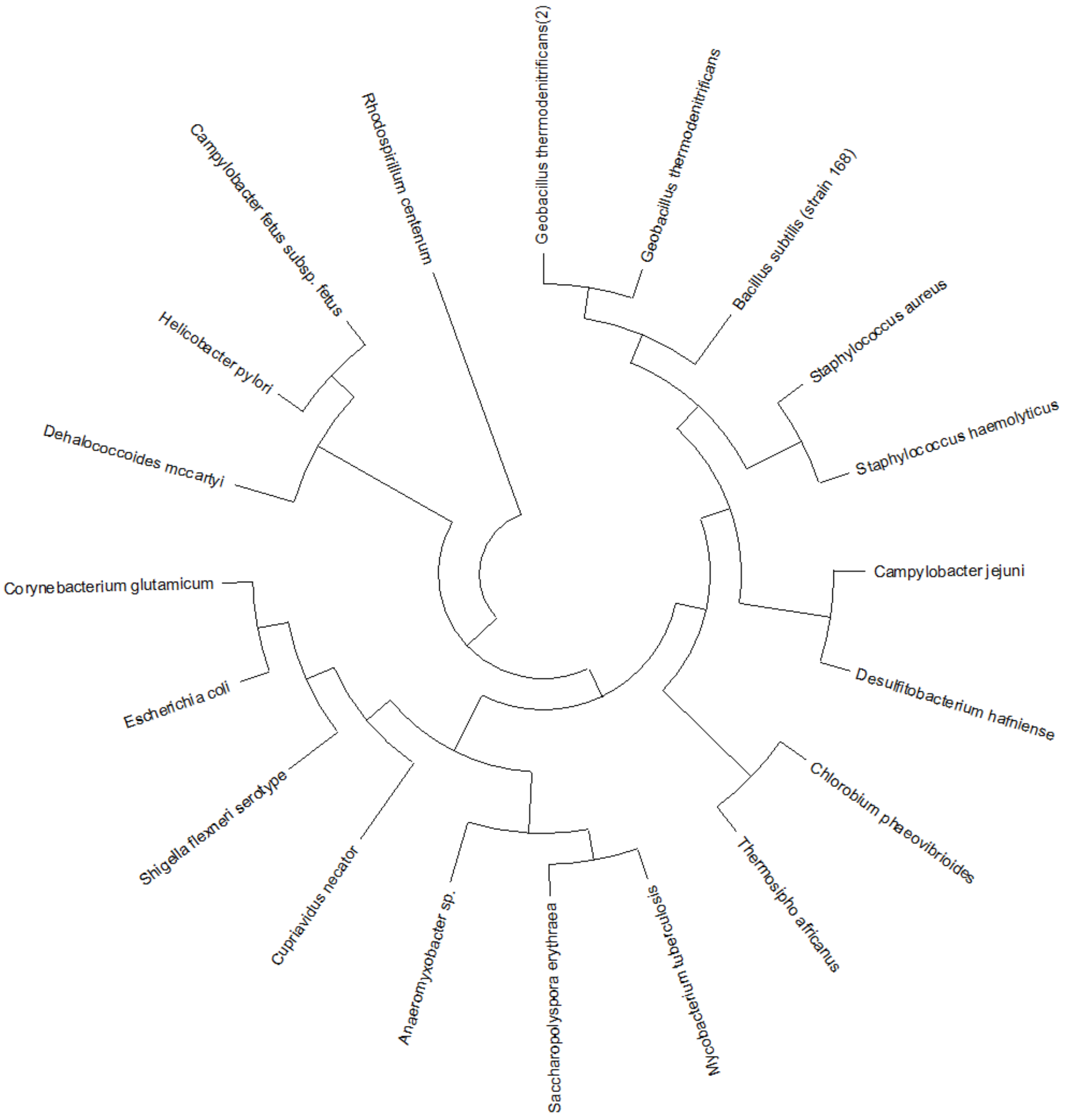

Figure S1. Phylogenetic tree of PanDs.

```
                     1        10        20        30        40        50        60
Shigella             MIRTMLQGKLHRVKVTHADLHYEGSCAIDQDFLDAAGILENEAIDIWNVTNGKRFSTYAI
Escherichia          MIRTMLQGKLHRVKVTHADLHYEGSCAIDQDFLDAAGILENEAIDIWNVTNGKRFSTYAI
Mycobacterium        MLRTMLKSKIHRATVTCADLHYVGSVTIDADLMDAADLLEGEQVTIVDIDNGARLVTYAI
Helicobacter         MTFEMLYSKIHRATITDANLNYIGSITIDEDLAKLAKLREGMKVEIVDVNNGERFSTYVI
Cupriavidus          MQRIMLRAKLHRVTVTQADLNYEGSCGIDQDLLDAADMKEFEKIELYNVNNGERFSTYII
Dehalococcoides      MLKSKIHRATITDACIDYEGSITIDKNLMQAANLLPYEQVHVVNVNNGTRLETYVIEGSA
Chlorobium           MKLHLLKSKIHNAIVTSGDLEYEGSITVDSELLKKADMIPNEKVLVVNNNNGERFETYII
Bacillus             MYRTMMSGKLHRATVTEANLNYVGSITIDEDLIDAVGMLPNEKVQIVNNNNGARLETYII
Campylobacter        MNITLLKSKIHRANVTEARLDYVGSISIDEKLLQASGILEYEKVQVVNVNNGARFETYTI
Staphylococcus       MIRTMMNAKIHRARVTESNLNYVGSITIDSDILEAVDILPNEKVAIVNNNNGARFETYVI
Saccharopolyspora    MFRTMLKSKIHRATVTQADLHYVGSVTVDADLMDAADLLEGEQVAIVDVTNGARLETYVI
Rhodospirillum       MIKVVRAKLHGITVTGADLNYHGSITLDPEICAQAGIYPMEFVEIWNRNSGARISTYVIF
Anaeromyxobacter     MRRTFFKAKIHRATVTHADLEYEGSVSIDEDLLEAAGIWEYEAVHIWNITRGTRLQTYAI
Thermosipho          MQEIMLKSKIHMAKVTDKSINYMGSIGIDTELLEKAGMKPYELVLVADVNNGQRFVTYII
Staphylococcus       MIRTMMNGKIHRARVTESNLNYVGSITIDADILDAVDILPNEKVAIVNNNNGARFETYVI
sp|A4IQ59|PAND_GEOTN MFRTLMNAKIHRARVTEANLDYVGSITIDEDILDAVGMVANEKVQIVNNNNGARFETYII
Geobacillus          MFRTLMNAKIHRARVTEANLDYVGSITIDEDILDAVGMVANEKVQIVNNNNGARFETYII
Campylobacter        MEIEMLYSKIHRATVTDANLNYVGSITIDERLMKAANLLEFQKVEILDINNGERFQTYVI
sp|Q251P1|PAND_DESHY MFRTMMKSKIHRATVTEANLKYVGSITIDEELLEVADILPNEKVQVVNNNNGARLETYVI
Corynebacterium      EFMIRTMLQGKLHRVKVTHADLHYEGSCAIDQDFLDAAGILENEAIDIWNVTNGKRFSTY

                              70        80        90       100       110       120
Shigella             AAERGSRIISVNGAAAHCASVGDIVIIASFVTMPDEEARTWRPNVAYFEGDNEMKRTAKA
Escherichia          AAERGSRIISVNGAAAHCASVGDIVIIASFVTMPDEEARTWRPNVAYFEGDNEMKRTAKA
Mycobacterium        TGERGSGVIGINGAAAHLVHPGDLVILIAYATMDDARARTYQPRIVFVDAYNKPIDMGHD
Helicobacter         LGKKRGEICVNGAAARKVAIGDVVIILAYASMNEDEINAHKPSIVLVDEKNEILEKG...
Cupriavidus          KGERGSGEISLNGAAARRAHLGDQLIICTYAPMSDEEIAAYKPKVILVNEKNGIKEIKKF
Dehalococcoides      GSGQICLNGAAARMGMKGDKIIILGYSLITEEDTLIHQPNLVYVDALNRITKVKNGVANQ
Chlorobium           KGDHGSRVIQLNGAAARCALPGDEIIIMTFCEIEAEEAPDFKPMVLIVDKNNNPKRRHRI
Bacillus             PGKRGSGVICLNGAAARLVQEGDKVIIISYKMMSDQEAASHEPKVAVLNDQNKIEQMLGN
Campylobacter        ATQEEGVVCLNGAAARLAEVGDKVIIMSYADFNEEEAKTFKPKVVFVDENNTATKITNYE
Staphylococcus       AGERGSGKICLNGAASRLVEVGDVVIIMTYAQLNEEEIQNHAPKVAVMNEDNVIIEMIHE
Saccharopolyspora    TGERGSGVIGINGAAAHLIEPGDLVILISYGVMDELEARSVRPKVIFVDADNRIVERGQD
Rhodospirillum       GEPGSGCCILNGAAARTCQRGDVIIIAASEYTTPDRLYTLKPRILTFTAGNRVDRVLRYD
Anaeromyxobacter     KGERGSGIICINGAAAHLNRPGDMVILATFAELEEAEARDFKPTVVLVDRQNKIVAKDAV
Thermosipho          PEEKGSKKIVINGAAARLVEQGDRVIIMAFGMYDSNEYKGPKVIIMNEKNEIVEIKQEGV
Staphylococcus       AGERGSGKICLNGAASRLVEVDDVVIIMTYVQLDERELVDHAPKVAVMNEKNEIIQMIHE
sp|A4IQ59|PAND_GEOTN PGERGSGVFCLNGAAARLVQKGDIIIVISYALVPEEKIAMHRPKVAIMDENNRIAQLIAE
Geobacillus          PGERGSGVFCLNGAAARLVQKGDIIIVISYALVPEEKIAMHRPKVAIMDENNRIAQLIAE
Campylobacter        KGEKKGEICLNGAAARKVCIGDIIIIVSYASMKRKKAKNFSPTIVHVNEKNEINE.....
sp|Q251P1|PAND_DESHY PGKRGERTVCLNGAAARLVQVGDEVIIIAYGIFTDEAARTYEPKVIFVDEGNNPVKIAHE
Corynebacterium      AIAAERGSRIISVNGAAAHCASVGDIVIIASFVTMPDEEARTWRPNVAYFEGDNEMKRTA

Shigella             IPVQVA.....................................
Escherichia          IPVQVA.....................................
Mycobacterium        PAFVPENAGELLDPRLGVG........................
Helicobacter         ...........................................
Cupriavidus          ...........................................
Dehalococcoides      GLEV.......................................
Chlorobium           GEEDEQLG...................................
Bacillus             EPARTIL....................................
Campylobacter        KHGAIF.....................................
Staphylococcus       KENTIVL....................................
Saccharopolyspora    PGHAPAGSGLAGTAASVTSAITEAAAETDDAAKLDALLQQPEH
Rhodospirillum       VFKGPDRAFDFRTLDITAGEDAAALEPVPILD...........
Anaeromyxobacter     EVPGPARRVTA................................
Thermosipho          ...........................................
Staphylococcus       KEGTIIK....................................
sp|A4IQ59|PAND_GEOTN EPAHTIL....................................
Geobacillus          EPAHTIL....................................
Campylobacter        ...........................................
sp|Q251P1|PAND_DESHY EIHGQQS....................................
Corynebacterium      KAIPVQVAKL.................................
```

Figure S2. Multiple sequence alignment results of PanDs.

2. References:

(1) Lambrughi, M.; Maršić, Ž. S.; Saez-Jimenez, V.; Mapelli, V.; Olsson, L.; Papaleo, E. Conformational Gating in Ammonia Lyases. *bioRxiv* **2019**. https://doi.org/10.1101/583088.

(2) Cheng, X.; Chen, X.; Feng, J.; Wu, Q.; Zhu, D. Structure-Guided Engineering of: Meso -Diaminopimelate Dehydrogenase for Enantioselective Reductive Amination of Sterically Bulky 2-Keto Acids. *Catal. Sci. Technol.* **2018**, *8* (19), 4994–5002. https://doi.org/10.1039/c8cy01426d.

(3) Yuan, S.; Chan, H. C. S.; Filipek, S.; Vogel, H. PyMOL and Inkscape Bridge the Data and the Data Visualization. *Structure*. 2016. https://doi.org/10.1016/j.str.2016.11.012.

(4) Yu, S.; Yao, P.; Li, J.; Feng, J.; Wu, Q.; Zhu, D. Improving the Catalytic Efficiency and Stereoselectivity of a Nitrilase from: Synechocystis Sp. PCC6803 by Semi-Rational Engineering En Route to Chiral γ-Amino Acids. *Catal. Sci. Technol.* **2019**, *9* (6), 1504–1510. https://doi.org/10.1039/c8cy02455c.

(5) Wang, J. B.; Lonsdale, R.; Reetz, M. T. Exploring Substrate Scope and Stereoselectivity of P450 Peroxygenase OleTJE in Olefin-Forming Oxidative Decarboxylation. *Chem. Commun.* **2016**, *52* (52), 8131–8133. https://doi.org/10.1039/c6cc04345c.
